# Cholera Prevention Training Materials for Community Health Workers, Haiti, 2010–2011

**DOI:** 10.3201/eid1711.110806

**Published:** 2011-11

**Authors:** Anu Rajasingham, Anna Bowen, Ciara O’Reilly, Kari Sholtes, Katie Schilling, Catherine Hough, Joan Brunkard, Jean Wysler Domercant, Gerald Lerebours, Jean Cadet, Robert Quick, Bobbie Person

**Affiliations:** Centers for Disease Control and Prevention, Atlanta, Georgia, USA (A. Rajasingham, A. Bowen, C. O’Reilly, K. Sholtes, K. Schilling, C. Hough, J. Brunkard, J. Cadet, R. Quick, B. Person); Oak Ridge Institute for Science and Education, Oak Ridge, Tennessee, USA (A. Rajasingham, K. Sholtes); Centers for Disease Control and Prevention, Port-au-Prince, Haiti (J.W. Domercant); Ministry of Public Health and Population, Port-au-Prince (G. Lerebours)

**Keywords:** bacteria, waterborne infections, cholera, Haiti, community health worker, public health, training, material development, ORS, oral rehydration solution, evaluation, dispatch

## Abstract

Stopping the spread of the cholera epidemic in Haiti required engaging community health workers (CHWs) in prevention and treatment activities. The Centers for Disease Control and Prevention collaborated with the Haitian Ministry of Public Health and Population to develop CHW educational materials, train >1,100 CHWs, and evaluate training efforts.

Because cholera can kill within hours of disease onset and access to cholera treatment centers is poor in many developing countries, community health workers (CHWs) are vital for educating community members about cholera transmission, prevention, and control and, when necessary, providing life-saving treatment. CHWs are typically laypersons selected by the community (*1**,**2*). For CHWs to effectively educate and support their communities during a cholera outbreak, they must be appropriately trained.

Cholera outbreaks typically arise in settings where water, sanitation, and hygiene infrastructures are inadequate (*3*). Even before Haiti was ravaged by an earthquake in January 2010, only 63% of the population had access to improved water sources, and only 17% had access to improved sanitation (*4*). Therefore, when the cholera outbreak began in October 2010, the Haitian Ministry of Public Health and Population (MSPP) was still actively developing and rebuilding the public health and water, sanitation, and hygiene infrastructures, and many communities were beyond the reach of these services. From the onset, MSPP initiated 3 strategies to enhance care of patients during the cholera epidemic: reinforcing existing heath care facilities with training and supplies, establishing a network of cholera treatment centers for management of severe cases, and mobilizing CHWs who could take treatment and prevention activities into the community.

The community-level strategy was particularly important in Haiti. A rapid assessment early in the outbreak indicated that among 87 cholera decendents, 39 (45%) died in the community, 60 (69%) did not suspect their illness was cholera or recognize the severity, 22 (26%) lived too far from health facilities to access care, and 30 (77%) of the 39 community decedents did not take oral rehydration solution (ORS) at home (J. Routh, pers. comm.). Populations with poor access to health care often experience higher case-fatality rates during cholera epidemics, which means that local provision of treatment and supplies through less specialized health workers such as CHWs is essential (*5**–**9*).

## The Study

To assist CHWs in conducting cholera education, prevention, and treatment activities, a multidisciplinary team that included physicians, behavioral scientists, epidemiologists, engineers, and communication specialists at the Centers for Disease Control and Prevention (CDC) and MSPP developed a set of technically accurate, lower literacy, and culturally adapted training and educational materials for CHWs during the cholera outbreak in Haiti. The materials are comprehensive in scope and can be adapted for use in cholera preparedness and response activities in other countries.

Training materials were based on the World Health Organization technical guidelines for cholera; guidelines and educational materials from the International Centre for Diarrhoeal Disease Research, Bangladesh; cholera prevention messages, guidelines, and materials from CDC; online manuals and guides from various nongovernmental organizations; and information from manufacturers of point-of-use water treatment products. Consensus for all materials was reached among CDC subject matter experts and communications personal, MSPP, and CDC staff in Haiti. Amendments were made after field review and use in Haiti, and messages concerning culturally appropriate burial practices, stigma prevention, and additional water treatment options were added as the epidemic progressed and additional content needs were identified.

**Figure 1:**
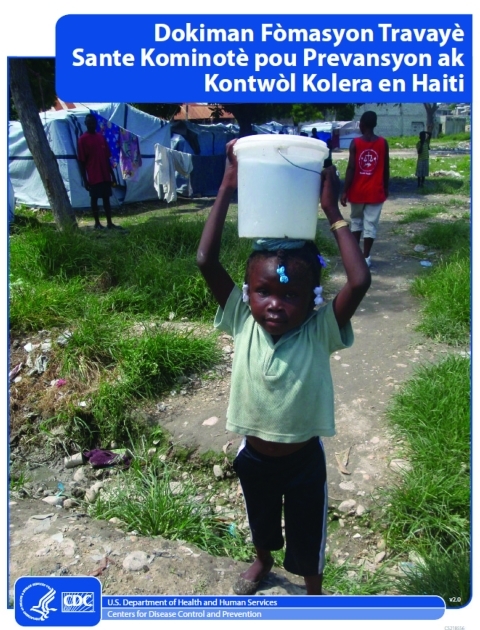
Cover page of community health worker cholera prevention and control training manual, Haiti, 2011.

**Figure 2:**
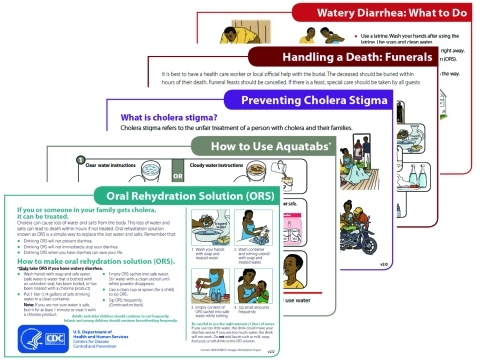
Series of community education cards developed for use in Haiti, 2011.

The materials now include comprehensive cholera prevention, treatment, and control training modules (Figure 1; Table 1, Table 2); a training guide and presentation slide set for use in teaching CHWs; and community education cards and low-literacy posters for CHWs to use within the community (Figure 2). Materials are available in Haitian Creole, French, and English.

**Table 1:** Summary of cholera prevention and control training materials developed for community health workers, Haiti, 2011*

Training Manual
Module 1: Community Mobilization
Module 2: What You Need to Know about Cholera
Module 3: Decision Making Guide for Taking Care of People with Watery Diarrhea
Module 4: Handwashing
Module 5: Oral Rehydration Solution (ORS)
Module 6: Safe Drinking Water: Aquatabs
Module 7: Safe Drinking Water: Dlo Lavi
Module 7A: Safe Drinking Water: Gadyen Dlo
Module 8: Safe Drinking Water: PuR
Module 9: Safe Water Storage
Module 10: Safe Food Preparation
Module 11: Safe Sanitation and Cleaning
Module 12: When a Person with Cholera Dies at Home
Module 13: Preventing Cholera Stigma
Community Education Cards
Community Mobilization
What You Need to Know about Cholera
Decision Making Guide for Taking Care of People with Watery Diarrhea
Handwashing
Oral Rehydration Solution (ORS)
Safe Drinking Water: Aquatabs
Safe Drinking Water: Dlo Lavi
Safe Drinking Water: Gadyen Dlo
Safe Drinking Water: PuR
Making Drinking Water Safe With Household Bleach
Safe Water Storage
Safe Food Preparation
Safe Sanitation and Cleaning
When a Person with Cholera Dies at Home
Cleaning after Flooding
Preventing Cholera Stigma
Training Guide
Optional PowerPoint presentation to accompany materials
Low-Literacy Posters
If You or Your Family Get Sick with Cholera (2 posters: Adult and Child versions)
How to Prepare Food Safely
Wash your Hands to Stop Cholera
How to Make and Use Oral Rehydration Solution (ORS)
How to Make Water Safe Using Aquatabs (5 posters, 1 for each dosage of Aquatab)
How to Make Water Safe Using Dlo Lavi
How to Make Water Safe Using Gadyen Dlo
How to Make Water Safe Using PuR
How to Make Water Safe Using Household Bleach
How to Practice Safe Sanitation and Cleaning
How to Prevent Cholera Stigma
How to Make a Tippy Tap

*These materials are available at www.cdc.gov/cholera/materials.html.

**Table 2:** Key cholera questions and response messages for community health workers, Haiti, 2011

What is cholera disease?
Cholera disease causes a lot of watery diarrhea and vomiting.
Cholera diarrhea can look like cloudy rice water.
Cholera can cause death from dehydration (the loss of water and salts from the body) within hours if not treated.
How is cholera spread?
Cholera germs are found in the feces (poop) or vomit of infected people.
Cholera is spread when feces (poop) or vomit from an infected person gets into the water people drink or the food people eat.
Cholera is not likely to spread directly from one person to another.
What are the key ways to protect yourself and your family from cholera and other diarrheal diseases?
Drink and use safe water. (Safe water is water that is bottled with an unbroken seal, has been boiled, or has been treated with a chlorine product.)
Wash hands often with soap and safe water. If no soap is available, scrub hands often with ash or sand and rinse with safe water.
Use latrines or bury your feces (poop), do not defecate in any body of water.
Cook food well (especially seafood), eat it hot, keep it covered, and peel fruits and vegetables.
Clean up safely— in the kitchen and in places where your family bathes and washes clothes.
What should you do if you or your family is ill with diarrhea?
If you have oral rehydration solution (ORS), start taking it now; it can save your life.
Go immediately to the nearest health facility, cholera treatment center, or community health worker, if you can.
Continue to drink ORS at home and while you travel to get treatment.
Continue to breastfeed your baby if they have watery diarrhea, even when traveling to get treatment.

As part of CDC’s response to the cholera outbreak in Haiti, this manual was made available through both CDC’s emergency website (www.cdc.gov/haiticholera) and cholera-specific website (www.cdc.gov/cholera). As of July 27, 2011, the websites had received >6,300 total views and 1,352 downloads. The training manual was also included as an appendix to CDC’s national clinical cholera training course for medical staff in Haiti, which was initiated on November 15, 2010.

CDC supported MSPP in organizing a Train-the-Trainer workshop March 1–3, 2011, in Port-au-Prince, Haiti. Twenty-four master trainers, including physicians, nurses, and other health care providers, were selected from various partner organizations and MSPP regional entities with large CHW cohorts. After the master trainers completed the Train-the-trainer course, they were asked to train CHWs within their own organizations in a cascading training approach. In June 2011, CDC conducted a preliminary follow-up evaluation of the Train-the-trainer course among master trainers to learn how it had contributed to subsequent trainings of CHWs. Participants included representatives from 9 departments and all partner organizations who attended the March Train-the-trainer workshop.

Survey questionnaires were completed by 14 of the 24 original Train-the-trainer participants. Among these, 8 participants reported training a total of 1,144 CHWs before the March Train-the-trainer course. Additionally, 10 participants reported training 1,170 CHWs in 9 departments of Haiti in the 3 months after the March Train-the-trainer event. Among the 10 participants who trained CHWs after the Train-the-trainer course, 9 reported using the CDC/MSPP CHW manual; 7 reported using the CDC/MSPP community education cards; and all demonstrated how to use soap, water treatment products, and ORS. Nine reported providing the CDC/MSPP manual to CHWs during trainings, 8 distributed water treatment products and ORS, and 7 distributed soap. All of those who trained CHWs after the March Train-the-trainer session reported that by the end of the training, CHWs were able to successfully demonstrate handwashing techniques, and most (9) indicated that CHWs were able to demonstrate proper preparation of safe water and ORS. In addition, 12 of the 14 participants who completed surveys reported training other types of community workers, including midwives, professors, group leaders, nurses, brigadiers, promoters, voodoo priests, and other religious leaders.

## Conclusions

In an attempt to quickly and efficiently reach the most underserved areas of Haiti during a deadly cholera outbreak, we developed comprehensive training materials for CHWs and implemented training using a Train-the-trainer model. However, this process had many challenges. The urgent need for training in Haiti required us to draft messages without the benefit of formative research or pilot testing of the materials. Translating materials into Haitian Creole, which only became an official language in Haiti in 1987 and which remains a predominantly verbal language with several dialects, was difficult and required multiple quality assurance steps. Because of resource constraints, Train-the-trainer events were not initiated in Haiti until March 2011, >4 months after the epidemic began. More timely training might have helped mitigate the impact of the epidemic on remote and underserved populations. These materials can now be adapted to train CHWs in other cholera-affected countries more rapidly than we were able to do in Haiti. Additional evaluation activities are planned at the community and household levels to assess the impact of these materials on cholera knowledge, prevention activities, and treatment among CHWs and community members and to guide revisions of these materials.

In many rural areas of Haiti, CHWs are the backbone of the health care system and can play an essential role in preventing cholera illness and death among medically underserved populations. We prepared standardized materials and training modules for CHWs that focused on prevention, treatment, and control of cholera. We conducted a Train-the-trainer workshop that led to training CHWs across 9 departments of Haiti. Additional monitoring and evaluation activities are needed to assess the reach and impact of the training materials and implementation.
